# Urolithin A‑producing *Limosilactobacillus fermentum* FUA033 fermentation significantly improves the sensory and antioxidant properties of strawberry juice

**DOI:** 10.1016/j.fochx.2025.102805

**Published:** 2025-07-16

**Authors:** Qing Wu, Wenyue Ma, Shu Liu, Fandi Zhou, Hao Wu, Xiangyuan Wang, Guang Yang, Yaowei Fang

**Affiliations:** a School of Ocean Food and Biological Engineering, Jiangsu Ocean University, Lianyungang 222005, China; b Co-Innovation Center of Jiangsu Marine Bio-Industry Technology, Jiangsu Ocean University, Lianyungang 222005, China; c The First Clinical Medical College, Nanjing University of Chinese Medicine, Nanjing 210000, China

**Keywords:** Strawberry juice, Lactic acid bacteria fermentation, Flavor volatile compounds, Sensory properties, Phenolic acid compounds

## Abstract

Probiotics, especially lactic acid bacteria-mediated fermentation, are recognized as an efficient approach to enhance the nutritional and functional properties of food matrices. In this study, *Limosilactobacillus fermentum* FUA033 was used to ferment strawberry juice, and its effects on bioactive compounds and sensory properties were evaluated. Sensory analysis was performed through the identification of volatile organic compounds (VOCs) and descriptive sensory evaluation. Fermentation significantly enhanced antioxidant activity and increased the total phenolic and flavonoid contents. Notably, the specific production of Urolithin A (Uro-A) by L. *fermentum* FUA033 reached a concentration of 10.27 ± 1.11 μM. Furthermore, fermentation altered the composition of phenolic acids and VOCs, resulting in improved sensory attributes and a significantly higher overall sensory score for the juice. Our current findings highlight the potential of L. *fermentum* FUA033 to enhance both the functional and flavor profiles of strawberry juice, supporting its application in the development of high-value functional foods.

## Highlights

• *L. fermentum* FUA033 improved sensory traits and flavor compounds in strawberry juice.
• *L. fermentum* FUA033 increased the Uro-A content in strawberry juice.
• *L. fermentum* FUA033 enhanced antioxidant capacities within strawberry juice.
• *L. fermentum* FUA033 altered the content and composition of phenolic acids.

## 1 Introduction

With an increasing understanding of healthy living and probiotic fermentation among people, the demand for functional foods is also growing (Sharma, Garg, Kumar, Bhatia, & Kulshrestha, 2020). Probiotic fermentation is a process that harnesses the metabolic activity of beneficial microorganisms and has been studied widely for its ability to improve the nutritional and bioactive properties of food products (Nuraida, 2015). By breaking down complex organic molecules into simpler, more bioavailable compounds, probiotic fermentation can significantly increase the contents of vitamins, phenolic compounds, amino acids, and extracellular polysaccharides in products. Additionally, this process has been shown to enhance the antibacterial, anticancer, antidiabetic, and anti-inflammatory properties of food products, making it a promising approach for functional food development (Brugnoli et al., 2023; Szutowska, 2020).

Berry fruits are regarded as processing substrates for probiotic fermentation due to their nutritional profile and excellent processing properties. Among various berry species, strawberries (Fragaria × Ananassa Duch.) exhibit favorable characteristics for microbial fermentation, including optimal tissue structure and superior nutrient bioavailability that facilitate efficient probiotic growth and metabolic activity (Aguilera, 2024; Seeram, 2008). Strawberries are among the most widely consumed berries worldwide (Basu, Nguyen, Betts, & Lyons, 2014; Filannino, Bai, Di Cagno, Gobbetti, & Gänzle, 2015; Septembre-Malaterre, Remize, & Poucheret, 2018). Strawberries contain abundant minerals, vitamins C and E, phenolic compounds, flavonoids, β-carotene, and folic acid, thereby providing plentiful substrates for microbial biotransformation (Peretto et al., 2014; Skrovankova, Sumczynski, Mlcek, Jurikova, & Sochor, 2015). Among these compounds, ellagitannins (ETs) and ellagic acid (EA) exhibit remarkable biological activity (Liu et al., 2023). However, the intrinsic bioavailability of the key bioactive ETs and EA in strawberries is limited. Interestingly, EA is metabolized by the gut microbiota to produce urolithins, including urolithin A (Uro-A), which exhibit significantly enhanced bioactivity and bioavailability (Hua, Liu, Yang, Hou, & Fang, 2024). Uro-A is particularly noted for its ability to improve cellular health by promoting mitochondrial autophagy and function while reducing inflammation (Hua et al., 2024). However, the natural conversion of EA to Uro-A is highly inefficient, due to which, chemical synthesis is used as the primary method for industrial-scale production of Uro-A (Xia et al., 2024). Chemical synthesis of Uro-A often results in residual organic solvents. Microbial fermentation, on the other hand, offers a more sustainable alternative with milder reaction conditions and the potential for natural product labeling. However, its industrial-scale application is not without challenges. Strain instability during prolonged fermentation can compromise Uro-A conversion efficiency, and issues such as contamination control and downstream processing must be carefully managed. Strategies to address these limitations include targeted genetic engineering of production strains, stringent quality control of raw materials, and enhanced contamination-prevention protocols to ensure robust and scalable fermentation. Consequently, microbial fermentation represents a better alternative to chemical synthesis for the industrial production of Uro-A (Sun et al., 2015).

Recent studies have reported that *Limosilactobacillus fermentum* FUA033 can metabolize EA into Uro-A, and this process may be utilized as a novel approach to increase the bioavailability of polyphenols in fermented food products. Therefore, this study aimed to evaluate the impact of fermentation with L. *fermentum* FUA033 on the physicochemical properties, bioactivities, and antioxidant capacity of strawberry juice. Additionally, this study employed headspace solid-phase microextraction coupled with gas chromatography–mass spectrometry (HS-SPME-GC–MS) to quantify phenolic acids and volatile compounds in the fermented juice. The results revealed that fermentation significantly enhanced the antioxidant capacity of strawberry juice and facilitated Uro-A conversion. In addition, various phenolic acids were detected after fermentation, and the flavor profile of the juice was also improved. These findings highlighted the potential of probiotic fermentation as a viable strategy for functional-beverage development and sustainable Uro-A production. Therefore, leveraging probiotic fermentation could revolutionize the development of bioactive-enriched functional beverages, thereby contributing to advancements in both food science and human health.

## 2 Experimental materials

### 2.1 Lactic acid bacterial strains

The Uro-A-producing strain L. *fermentum* FUA033, which was originally obtained from a human intestinal isolate collected by the Jiangsu Key Laboratory of Marine Biotechnology and then deposited in the China General Microbiological Culture Collection Center (CGMCC No. 28447), was used in this study.

### 2.2 Materials

Fresh strawberries to be used for juice preparation in this study were purchased from a commercial grower in Lianyungang, Jiangsu Province, China. The sampling geographic coordinates are 34°39′57.3”N 118°53′00.8″E. All fruits were harvested at a uniform ripeness stage, identified by full red coloration and a Brix value of 8.5–9.5 %. Only fully ripe and undamaged strawberries were selected for use. Analytical-grade EA and Uro-A standards were supplied by Sigma-Aldrich (USA). HPLC-grade formic acid, acetonitrile, and methanol were purchased from the same vendor. The DPPH, total antioxidant capacity, and hydroxyl-radical assay kits were purchased from Nanjing Jiancheng Bioengineering Institute (China). DeMan-Rogosa-Sharpe media for bacterial cultivation were purchased from Thermo Fisher Oxoid (UK). The key instruments used included an Agilent 1260 HPLC system (Agilent Technologies, Germany), an FE28 pH meter (Mettler-Toledo, Switzerland), an A51119600DPC microplate reader (Thermo Fisher Scientific, USA), and an A20 anaerobic workstation (Don Whitley Scientific, UK).

## 3 Experimental methods

### 3.1 Strawberry juice preparation

Fresh strawberries were put in deionized water at 25 °C for 8 h and then pulped. The pulp was centrifuged at 4000 ×*g* for 5 min to obtain the supernatant, which served as the juice sample. The juice was pasteurized at 80 °C for 15 min before use in the subsequent fermentation trials.

### 3.2 Fermentation of strawberry juice with L. *fermentum* FUA033

*L. fermentum* FUA033 was cultured in Man-Rogosa-Sharpe (MRS) medium at 37 °C for 24 h to achieve optimal growth. The bacterial culture was then mixed with sterile glycerol at a 25 % volume ratio to form a glycerol suspension, which was stored at −80 °C for long-term preservation to ensure strain viability and stability of the strain.

For the fermentation experiments, the L. *fermentum* FUA033 strain was retrieved from the −80 °C storage and cultured in MRS medium for activation to a suitable cell density (5 × 10^7^ CFU mL^−1^). A 2 % (*v*/v) inoculum of the activated culture was added to 100 mL of the sterilized strawberry juice sample, followed by incubation at 37 °C for 48 h under static conditions. The composition of the MRS liquid medium was as follows: tryptone (1 %), beef extract (1 %), yeast extract (0.5 %), and glucose (2 %). The medium was sterilized at 121 °C for 20 min and then cooled to room temperature before use. This medium was used for the activation and cultivation of *Lactobacillus plantarum* prior to subsequent fermentation experiments.

### 3.3 Determination of the physicochemical indicators

After fermentation, the viability of the probiotic strains was determined using the plate count technique as described by Nemo and Bacha (Nemo & Bacha, 2020). The pH of the fermented strawberry juice was recorded using a digital pH meter (FE28, Mettler Toledo, China). The total sugar content was expressed as grams per 100 mL. The amounts of total phenolics and flavonoids were assessed using the Folin-Ciocalteu reagent and the aluminum chloride colorimetric assay, respectively.

### 3.4 Detection of the phenolic acid compounds

Phenolic acids in the fermented and unfermented juice samples were analyzed using an ACQUITY UPLC system (Agilent, USA) equipped with a BEH C_18_ column (2.1 × 100 mm, 1.7 μm; Waters Ltd., MA, USA). Chromatographic separation was performed at 0.3 mL/min using a binary mobile phase consisting of solvent A (0.1 % formic acid in water) and solvent B (0.1 % formic acid in acetonitrile). The gradient program was initiated with 5 % B, ramped to 95 % within 0.5 min, held for 2 min (11.0–13.0 min), and then returned to initial conditions. The column temperature was maintained at 30 °C.

Detection was performed at 305 nm using a photodiode array detector. Quantification was achieved using the external standard method with calibration curves constructed from authentic phenolic acid standards, including gallic acid, caffeic acid, p-coumaric acid, and ferulic acid (Sigma-Aldrich, USA). Calibration curves were linear over the tested concentration ranges (R^2^ > 0.99). All analyses were conducted in triplicate.

### 3.5 Antioxidant activity assessment

DPPH radical scavenging activity was assessed using a modified procedure from Li et al. (Li, Ji, Yang, & Lu, 2019). Briefly, 0.1 mL of the test sample was added to 3.9 mL of a DPPH methanol solution (0.025 g/L) and left in the dark at ambient temperature for 30 min. After the reaction, absorbance was measured at 515 nm using a UV–Vis spectrophotometer (Shimadzu, Japan). A blank containing 0.1 mL of ethanol was used instead of the sample in the control experiments. The percentage of DPPH radical scavenging was calculated according to the following equation.

$$\text{DPPH}\;\mathrm{RSA}\;\left(\%\right)=\frac{A_{\text{control}}-A_{\text{sample}}}{A_{\text{control}}}\times 100\%$$

ABTS radical cation decolorization (ABTS^+^)-scavenging activity assay.

The ABTS^+^ radical scavenging capacity was evaluated following the method of Shan et al. (Shan, Cai, Sun, & Corke, 2005), in which ABTS is oxidized to generate stable ABTS^+^ cation radicals. A solution of 7 mM ABTS and 2.45 mM potassium persulfate was incubated in the dark at 20 °C for 24 h to allow for radical formation. Prior to use, the ABTS^+^ solution was diluted with ethanol to an absorbance of 0.700 ± 0.020 at 734 nm. Next, 10 μL of the sample was mixed with 70 μL of distilled water and 4 mL of the diluted ABTS^+^ solution. After exactly 6 min of incubation at room temperature, the absorbance was measured at 734 nm (R. Kaprasob, Kerdchoechuen, Laohakunjit, Thumthanaruk, & Shetty, 2018).

Hydroxyl radical scavenging activity assay.

Hydroxyl radical scavenging activity assay (Hydroxyl RSA) was performed using a reaction mixture containing 1 mL of 1,10-phenanthroline (0.75 mM), 1.5 mL of sodium phosphate buffer (0.15 M, pH 7.4), 1 mL of FeSO_4_ (0.75 mM), 1 mL of H_2_O_2_ (0.01 %, *v*/v), and 1 mL of cell-free culture supernatant. The mixture was incubated at 37 °C for 30 min, and the absorbance was measured at 536 nm using a spectrophotometer (Thermo Fisher Scientific). The result value was calculated as follows:

$$\text{Hydroxyl}\;\mathrm{RSA}\;\left(\%\right)=\frac{A_{\text{test}}-A_{\text{blank}}}{A_{0}-A_{\text{blank}}}\times 100\%$$

### 3.6 Uro-A detection

The Uro-A content was measured using a Waters Xevo G2-XS quadrupole time-of-flight ultra-performance liquid chromatography-mass spectrometry system (QToF UPLC-MS). Separation was achieved on a BEH C_18_ analytical column (2.1 × 100 mm, 1.7 μm) at a flow rate of 0.3 mL/min. The binary mobile phase comprised 0.1 % formic acid in water (Solvent A) and 0.1 % formic acid in acetonitrile (Solvent B). Elution was performed using 5 % Solvent B for 0.5 min, followed by a linear increase to 95 % Solvent B over 11 min and maintenance at this concentration for an additional 2 min.

Mass spectral detection was carried out in the negative ionization mode across the *m*/*z* range of 50–1200. Leucine-enkephalin (m/z 554.2615) served as the internal standard for mass calibration. The ion source conditions were as follows: a capillary voltage of 2.5 kV, a cone voltage of 40 V, a source temperature of 121 °C, and a desolvation gas temperature of 400 °C. Mass Lynx 4.1 software was used for data collection and analysis.

### 3.7 Determination of the contents of volatile organic compounds

Volatile compounds were separated on a DB-5MS capillary column (30 m × 0.25 mm × 0.25 μm; Agilent & W Scientific, Folsom, CA, USA) using ultrahigh-purity helium (≥99.999 %) as the carrier gas at a fixed flow rate of 1.2 mL/min. The injection port was maintained at a temperature of 250 °C under split-less conditions, and a solvent delay of 3.5 min was applied. The oven program was as follows: 40 °C (held for 3.5 min), increased at 10 °C/min to 100 °C, then at 7 °C/min to 180 °C, and finally at 25 °C/min to reach 280 °C, where it was held constant for 5 min.

Mass spectral analysis was conducted using an electron impact (EI) ionization source operating at 70 eV. The source, quadrupole, and interface temperatures were set at 230 °C, 150 °C, and 280 °C, respectively. The selected ion monitoring (SIM) mode was used for compound identification and quantification in accordance with the GB 23200.8–2016 precision scanning guidelines.

### 3.8 Quantitative sensory evaluation

A quantitative descriptive sensory evaluation was performed based on the method outlined by Tao et al.(Tao & Cho, 2020), with minor modifications. A panel of ten trained assessors (five females and five males, aged 18–22), all specializing in Food Science and Technology, was assembled to carry out the descriptive analysis.

Before the formal assessment, the panelists engaged in a one-hour discussion session and a subsequent one-hour training session. During the discussion, a sensory lexicon was collaboratively developed using two reference samples: unfermented and fermented strawberry juice. The training phase then reinforced the consistent application of these descriptors through repeated exposure to the same reference samples.

Each panelist received six samples and evaluated them based on seven sensory attributes in an isolated sensory evaluation room. The assessment focused on four major categories: appearance (brightness, browning degree), aroma (fruity, acides), taste (sweetness, sourness), and overall acceptability, which has previously been identified as the most critical sensory attribute in fruit juice evaluation (Ren et al., 2015). The intensity and liking of each attribute were rated on a 10-point scale: 1–2 indicated very weak intensity or strong dislike; 3–4 reflected weak intensity or slight dislike; 5–6 represented moderate intensity or neutrality; 7–8 indicated strong intensity or moderate liking; and 9–10 reflected very strong intensity or strong liking. Sensory evaluation results for each attribute were calculated based on the average scores of 10 panelists and visualized using a sensory radar chart.

### 3.9 Statistical analysis

Statistical analyses and data visualization were conducted using GraphPad Prism (v10.1.2) and OriginPro (v2021), respectively. Variables that conformed to a normal distribution were expressed as mean values accompanied by standard deviations. Group differences were assessed through one-way analysis of variance (ANOVA), whereas pairwise comparisons were conducted using either the student's *t*-test or the Wilcoxon rank-sum test as suited for the data. A threshold of *p* < 0.05 was adopted to determine statistical significance throughout the analyses. Each experimental condition was tested in triplicate to increase data reliability and ensure reproducibility.

## 4 Results and discussion

### 4.1 Physicochemical properties during strawberry juice fermentation

During fermentation, the physicochemical properties of strawberry juice undergo significant changes, under the influence of microbial activity and biochemical transformation. The fermentation process can increase the bioavailability of phenolic compounds and modify the contents of organic acids, sugars, and volatile compounds, ultimately affecting the sensory and functional properties of the final product.

**Fig. 1:**
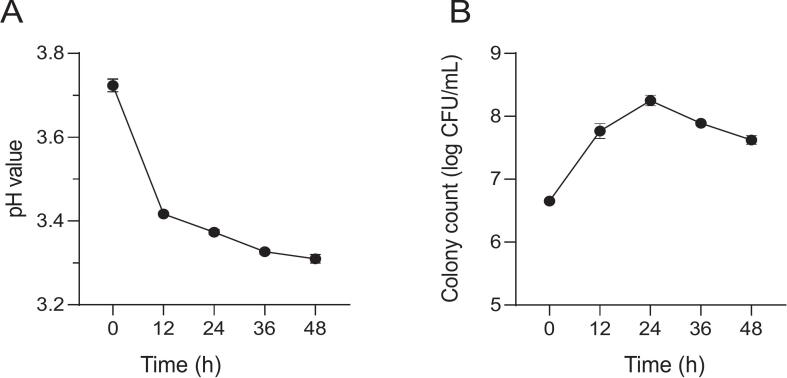
Physicochemical properties during strawberry juice fermentation. (A) pH values. (B) Colony counts.

The strain used in this study exhibited accelerated proliferation and growth in the presence of adequate nutrients, which resulted in significant alterations in its biological activities. This study analyzed the changes in pH and colony count density during strawberry juice fermentation, Fig. 1 showed the pH changes in strawberry juice before and after fermentation were measured (Fig. 1A). The initial pH of the juice was 3.72 ± 0.02(at 0 h), which decreased to 3.42 ± 0.01 within the first 12 h and then gradually to 3.31 ± 0.01(at 48 h) *(p* > 0.05), representing a total reduction of 12.3 %. This decrease can be attributed to the production of various organic acids during fermentation. These acids are generated when the bacterial strain metabolizes the naturally occurring soluble sugars and carbohydrates in the juice, which serve as essential substrates for bacterial growth and energy production.

Moreover, the resulting acidic environment creates optimal conditions for the proliferation of probiotics, which further enhances the functional properties of the fermented juice (Fazilah, Ariff, Khayat, Rios-Solis, & Halim, 2018).

Fig. 1. showed the colony abundance, highlighting that the survival of L. *fermentum* FUA033 was strongly influenced by medium composition and storage conditions. The microbial population served as a reliable indicator of the strain's adaptation to the fermentation environment. A significant increase in cell density was observed, from 6.65 ± 0.05 log CFU/mL to a peak of 8.25 ± 0.08 log CFU/mL. After 24 h, a slight decline occurred, although the final count remained significantly greater than the initial level (*p* < 0.01). These findings confirmed the strain's ability to thrive in strawberry juice, supporting the potential of this strain for probiotic beverage production (Muhialdin, Hussin, Kadum, Hamid, & Jaafar, 2021).

**Fig. 2:**
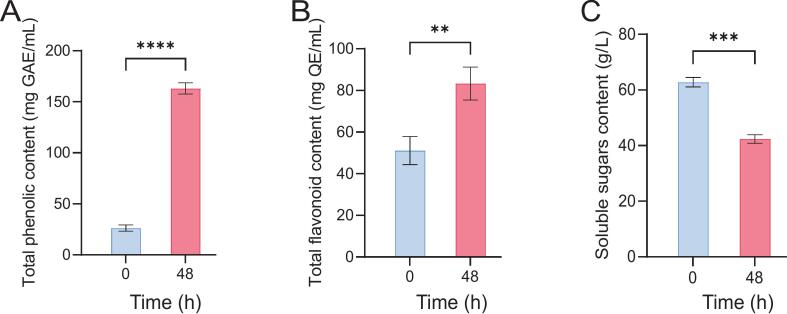
Changes in the phytochemical concentractions of strawberry juice during fermentation. (A) Total phenolic content(TPC). (B) Total flavonoid content(TFC). (C) Soluble sugars content(SSC). Data are presented as the mean ± SD (*n* = 3 per group). *p < 0.05, **p < 0.01, ***p < 0.001, ****p < 0.0001.

The above finding was consistent with the recommended cell count threshold of 6.00 log CFU/mL for probiotic beverages (Sheehan, Ross, & Fitzgerald, 2007), suggesting that L. *fermentum* FUA033 exhibited robust growth and effectively utilized the nutrients in strawberry juice to support its proliferation. It was accordingly hypothesized that after 48 h of fermentation, bacterial growth, and acid production would decline due to the increasingly acidic environment. However, in the experiments, the strain demonstrated resilience by maintaining its metabolic activity, including the production of enzymes that facilitated the release of bound flavonoids in strawberry juice. Fig. 2B showed the total flavonoid content (TFC) in the fermented strawberry juice (at 48 h) was significantly greater than that in the unfermented juice (at 0 h), supporting the above hypothesis.

In the literature, Bagher Hashemi et al. (Hashemi & Jafarpour, 2020) reported a rapid increase in bacterial density after 72 h of fermentation when *Lactobacillus plantarum* was used to ferment bergamot juice. Hai-Li Zhou et al. (Zhou et al., 2023) also reported a comparable trend in the fermentation of prickly pear with L. *fermentum* NCU001464, with bacterial densities exhibiting an initial increase before declining. This decline was attributed to the stressful effects of the acidic environment created due to LAB fermentation, which ultimately inhibited further bacterial growth.

### 4.2 Phytochemical concentrations during strawberry fermentation

The phenolic and flavonoid compounds in fruits and vegetables are crucial for human health, offering potential benefits such as reducing cancer risk, anti-inflammatory properties, and inhibition of carcinogenesis (de Souza, de Albuquerque, Dos Santos, Massa, & de Brito Alves, 2019). In this study, the fermentation of L. *fermentum* FUA033 led to a significant increase in the total phenolic content (TPC) and total flavonoid content (TFC) in strawberry juice. Specifically, the TPC increased from 26.16 ± 3.10 mg GAE/100 mL (at 0 h) to 163.13 ± 5.56 mg GAE/100 mL (at 48 h) (*p* < 0.001) (Fig. 2A). Similarly, the TFC increased from 51.16 ± 6.75 mg QE/100 mL (at 0 h) to 83.32 ± 7.86 mg QE/100 mL (at 48 h) (*p* < 0.01) (Fig. 2B).

Moreover, the soluble sugar content (SSC) markedly decreased from 62.78 ± 1.70 g/L to 42.35 ± 1.54 g/L, accounting for a reduction of 32.54 % over the fermentation period (*p* < 0.001) (Fig. 2C). These results suggested that lactic acid fermentation facilitates cell wall degradation in *Lactobacillus* strains through enzymatic activities and the creation of acidic environments. This process promotes the release of phenolic compounds and their conversion into more extractable forms, resulting in a marked increase in total phenolic content.Through quantitative analysis of phenolic acids in strawberry juice before and after fermentation, we observed significant compositional changes (Table 1). the major phenolic acids identified in unfermented strawberry juice included 3,4-dihydroxybenzoic acid, citric acid, and 4-hydroxybenzoic acid. Following fermentation with L. *fermentum* FUA033, the contents of chlorogenic acid 3-glucoside, citric acid, ferulic acid, butyric acid, cyanidin 3-O-rutinoside, and 3,5-dimethylbenzaldehyde increased. In contrast, the levels of benzoic acid, caffeic acid, 3,4-dihydroxybenzoic acid, 4-hydroxybenzoic acid, 4-hydroxycinnamic acid, ellagic acid, citric acid, and shikimicidin 3-O-rutinoside decreased. Notably, citric acid and mangiferic acid were undetectable before fermentation but exhibited a significant increase after 48 h of fermentation. Additionally, previously undetected acids, including mangiferic acid, ferulic acid, gallic acid, p-coumaric acid, vanillic acid, cornflower 3-O-glucoside, iridoidin 3-O-glucoside, and curcuminoid chloride, were identified post-fermentation.

**Table 1:** Phenolic and organic acids identified in strawberry juice before and after fermentation (0 h and 48 h).

**NO.**	**Compounds** **Name**	**Retention time**	**Peak area**		**m/z**
		**(min)**	**0H**	**48H**	
1	Malic acid	0.669	–	1,131,426	133.0143
2	Benzoic acid	3.898	35,434	27,356	121.0295
3	Caffeic acid	1.548	48,920	29,656	179.035
4	3,4-Dihydroxybenzoic acid	3.901	988,928	815,267	153.0193
5	4-Hydroxybenzoic acid	3.014	219,995	159,780	137.0244
6	p-Hydroxy-cinnamic acid	1.867	190,101	159,860	163.0401
7	Ellagic acid	0.973	46,225	9728	300.999
8	Pelargonidin-3-glucoside chloride	3.314	44,673	57,742	467.0751
9	Citric acid	0.645	3,223,423	3,235,839	193.0343
10	Shikimic acid	0.857	73,191	48,651	175.0601
11	Ferulic Acid	8.193	5110	11,031	195.0652
12	Gallic acid	0.706	–	24,647	171.0288
13	p-Coumaric acid	4.028	–	75,424	165.0546
14	Vanillic acid	0.859	–	18,957	169.0495
15	Syringic acid	0.879	35,461	65,285	199.0601
16	4-Hydroxy-3,5-dimethoxycinnamic acid	0.911	20,946	20,935	225.0758
17	Cyanidin 3-O-glucoside	8.558	–	13,290	485.0845
18	cyanidin 3-O-rutinoside	3.17	83,715	110,923	596.1736
19	Irigenin 3-O-glucoside	14.76	–	13,090	501.0794
20	Kuromanin chloride	8.558	–	13,290	485.0845
21	methylheptenone	7.666	30,281	29,335	127.1117
22	3,5-Dimethylbenzaldehyde	10.913	31,899	39,196	135.0804
23	3-Methylindole	3.152	10,429	10,052	132.0808
24	2-Methylbutyrate	8.712	–	6774	248.0917

The above finding aligns with the previous studies on L. *fermentum* fermentation in other plant-derived substrates, such as ginkgo almond juice (Y. Wang et al., 2019), mulberry juice (Kwaw et al., 2018), and sea buckthorn-apple juice (Tkacz, Chmielewska, Turkiewicz, Nowicka, & Wojdylo, 2020). For example, Ghosh et al. (Ghosh et al., 2015) reported an increase in TPC when rice beverages were fermented with L. *fermentum* KKL1, where the TPC increased from 11.80 mg GAE/g in the unfermented rice to 63.42 mg GAE/g post-fermentation. Similarly, fermentation with *Lactobacillus plantarum* MCC 2974 resulted in increased TPC and TFC levels in the Sohiong juice (Vivek, Mishra, Pradhan, & Jayabalan, 2019).

After 48 h of fermentation, the final pH of the strawberry juice reached 3.30, and although the colony density decreased, the cell count remained above the recommended threshold for probiotic functional beverages (Sheehan et al., 2007). These findings suggested that fermentation effectively promotes L. *fermentum* proliferation, demonstrating its potential for commercial applications. However, further research is needed to validate its functional properties and antioxidant capacity. To address this, antioxidant activity was measured in this study for additional verification.

Although total phenolic and flavonoid contents were measured as indicators of potential functional improvement, these alone are insufficient to support claims regarding bio-accessibility,our future studies will focus on evaluating bioavailability and functional activity through in vitro digestion models and relevant assays.

### 4.3 Analyses of the antioxidant activity of fermented berries

In recent years, berries have been recognized as “super fruits” because of their rich composition of bioactive compounds, including phenolic acids, flavonoids, anthocyanins, tannins, and ascorbic acid. These compounds have gained significant importance in the nutraceutical and functional food markets (Huang, Zhang, Liu, & Li Huang, Zhang, Liu, & Li, 2012), particularly in North America and Europe, where their antioxidant properties are being increasingly regarded as essential food quality indicators. According to Wang, Cao, and Prior (da Silva Pinto, Lajolo, & Genovese, 2008; H. Wang, Cao, & Prior, 1996), strawberries exhibited superior antioxidant capacity compared to other fruits, with substantial total antioxidant potential that effectively neutralized free radicals and mitigated oxidative stress in human cells (Afrin et al., 2016).

**Fig. 3:**
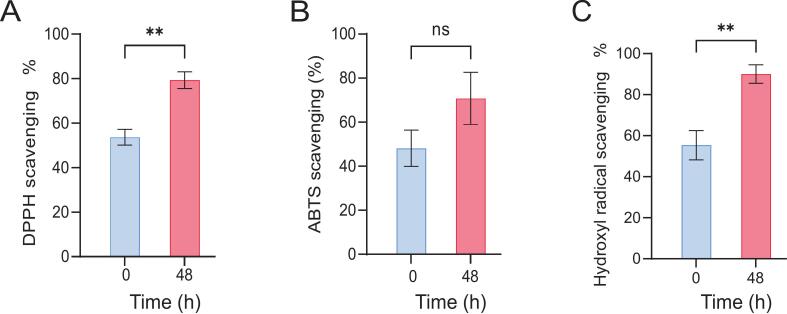
Antioxidant activity of strawberry juice during fermentation. (A) DPPH radical scavenging activity. (B) ABTS+ radical scavenging activity. (C) Hydroxylradical scavenging activity. The data are presented as the mean ± SD (n = 3 per group), *p < 0.05, ns:not significant.

In this study, the antioxidant activity of fermented strawberry juice was evaluated using ABTS, DPPH, and Hydroxyl radical scavenging assays. The results revealed a significant increase in the antioxidant function following fermentation. Specifically, the DPPH radical scavenging activity increased from 53.67 % to 79.33 % (*p* < 0.001) post-fermentation (Fig. 3A). This finding suggested that lactic acid bacterial fermentation increased the bioavailability of polyphenolic compounds with proton-donating properties (dos Santos Filho et al., 2019), thereby improving the DPPH radical scavenging capacity. Similarly, the ABTS radical scavenging activity and hydroxyl radical scavenging activity increased from 49.38 % and 55.33 %, respectively, to 69.58 % and 91.00 %, respectively, although these increases were not statistically significant (Fig. 3B and C). These trends were consistent with the observed increases in TPC and TFC discussed previously in this report (Fig. 2A and B), indicating a strong positive correlation between free radical scavenging capacity and the presence of phenolic compounds such as gallic acid, rutin, and populin. Among these compounds, ellagitannins accounted for 30 %–60 % of the total antioxidant capacity (Beekwilder et al., 2005) due to their strong electron-withdrawing properties, enabling free radical neutralization through resonance stabilization. Similar findings have been reported for the lactic acid fermentation of blueberry juice (Wu et al., 2021).

The enhancement of antioxidant properties through probiotic fermentation was further substantiated in this study. During fermentation, glycosidases hydrolyze the bound polyphenolic compounds (e.g., esterified phenolics), leading to an increased concentration of free phenolics and consequently a greater antioxidant potential. This enhanced bioavailability might also stimulate the body's intrinsic antioxidant defense mechanisms. Furthermore, Lactobacillus cells possess phenolic acid reductase, an intracellular antioxidant enzyme that is more potent than caffeic acid. The enzymatic breakdown of complex phenolic compounds into simpler phenols or their derivatives, such as caffeic acid derivatives, further contributes to the enhanced antioxidant efficiency.

In summary, fermentation significantly increased the antioxidant capacity of strawberry juice, reinforcing the findings of previous studies (Martín-Gómez, Varo, Mérida, & Serratosa, 2020). The superior antioxidant activity observed in the fermented strawberry juice compared to its unfermented counterpart suggested potential health benefits. These findings provide valuable insights and lay a theoretical foundation for the development of fermented fruit juices for use as functional foods.

### 4.4 Conversion of EA to Uro-a in fermented strawberry juice

EA is a naturally occurring polyphenol found in fruits, which has garnered significant attention from the scientific community because of its well-documented chemopreventive and antioxidant properties. In plants, EA exists in various derivative forms, each with different solubilities, mobilities, and reactivities across different biological systems. However, the bioavailability of EA in humans is limited because of its low solubility. Upon ingestion, EA undergoes microbial metabolism in the gut, producing urolithins, among which Uro-A (Sun et al., 2015; Yaling et al., 2024) is the most biologically active. Uro-A has been clinically proven to enhance muscle function through mitochondrial autophagy. It also exhibits a range of beneficial effects, including anticancer, anti-inflammatory, and immune-boosting properties, along with potential cognitive benefits, while no risks to human health are reported.

The metabolism of urolithins in humans can be classified into three phenotypes based on the ability to convert EA into specific metabolites: Uro-A, Uro—B, and Uro—C. However, only about 40 % of the population has the gut microbiota capable of converting EA into Uro-A, due to which the natural production of Uro-A is relatively uncommon (Liu et al., 2023).

Therefore, chemical synthesis remains the predominant method for large-scale Uro-A production to date. However, microbial fermentation, which operates under milder reaction conditions and is more scalable for industrial applications, has emerged as a promising alternative for Uro-A production. Given its potential to replace conventional chemical synthesis, microbial fermentation has attracted considerable interest in the nutraceutical and functional food industries.

The metabolic conversion of EA to Uro-A involves a series of enzymatic reactions, with the hydrolysis of ester groups as the first step. The produced hexahydroxydibenzoic acid undergoes a lactone ring opening under lactonase decarboxylase activity, yielding potential intermediate compounds. The action of specific dehydrogenases leads to the formation of UM5, which is subsequently converted to UM6 by 4-dehydroxylase. Further metabolic steps involve the transformation of UM6 to UC by 10-dehydroxylase, and, ultimately, the conversion of UC to Uro-A by 9-dehydroxylase.

**Fig. 4:**
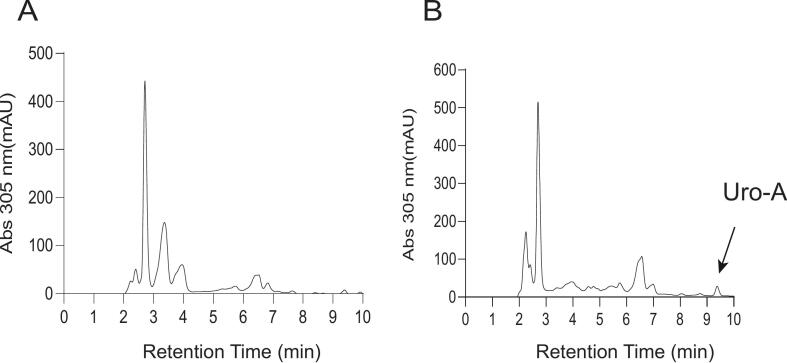
Detection of compound Uro-A in strawberry juice before and after fermentation. (A) HPLC chromatogram of unfermented juice extract. (B) HPLC chromatogram of fermented juice extract.

Previous studies have demonstrated that L. *fermentum* FUA033 can convert EA into Uro-A (Z. Wang et al., 2025). To evaluate this metabolic process in strawberries, this study employed UPLC-MS analysis to assess the transformation of EA to Uro-A during fermentation. The results revealed a gradual conversion over 48 h, culminating in a final Uro-A concentration of about 10.27 ± 1.11 μM (Fig. 4). This significant increase was attributed to the microbial metabolic activity during fermentation, which facilitates the release and transformation of bioactive compounds, thereby increasing the Uro-A content.

The findings of this study demonstrated that fermentation substantially increases the Uro-A concentration in strawberry juice, providing strong evidence in support of the effectiveness of fermentation in enriching the functional properties of fruit-based beverages. The findings offer valuable insights for the development of functional fermented foods with potential health benefits, paving the way for future research and commercial applications in this regard.

### 4.5 Identification of VOCs

The dynamic transformation of volatile compounds during strawberry juice fermentation plays a crucial role in determining a product's sensory characteristics. To comprehensively assess these transformations, HS-SPME-GC–MS was used in this study to identify the key metabolites, while principal component analysis (PCA) and hierarchical clustering heatmap analysis were applied to evaluate the shifts in the volatile compound profiles before and after fermentation.

**Fig. 5:**
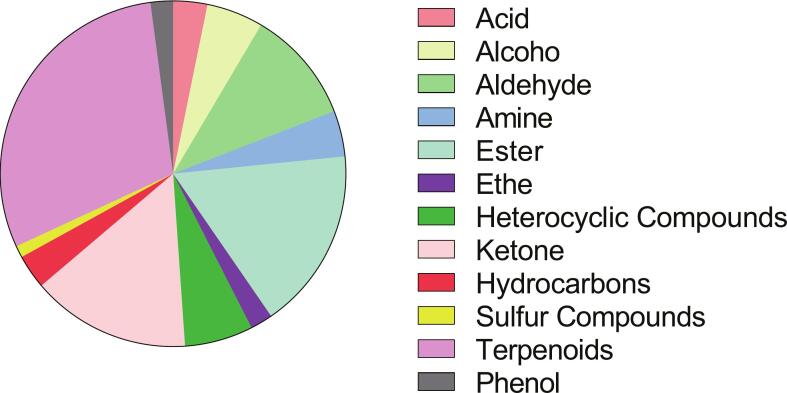
Identification of volatile metabolites by HS-SPME-GC–MS during fermentation of strawberry juice.

**Fig. 6:**
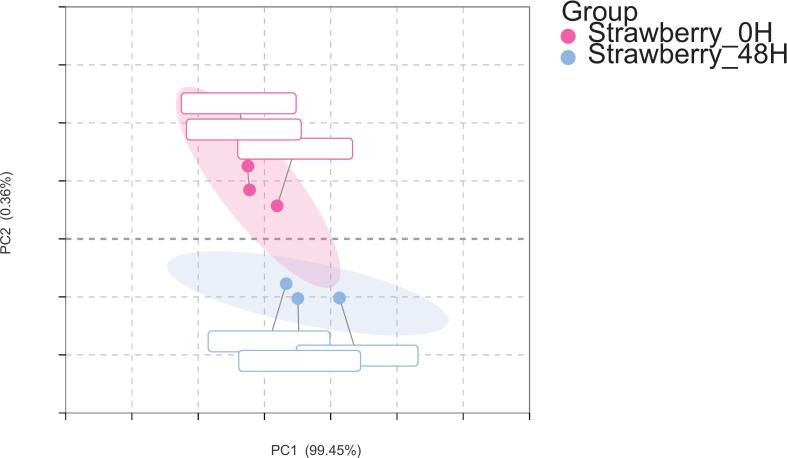
PCA plot based on the concentration changes of volatile components during the fermentation.

A total of 94 volatile metabolites were identified, spanning 12 chemical classes of acids (3.19 %), alcohols (5.32 %), aldehydes (10.64 %), amines (4.26 %), esters (17.02 %), ethers (2.13 %), heterocyclic compounds (9.57 %), hydrocarbons (3.19 %), phenols (2.13 %), sulfur-containing compounds (1.06 %), terpenes (29.79 %), and ketones (14.89 %) (Fig. 5). Fig. 6 showed PC1 accounted for 99.45 % of the total variance, whereas PC2 contributed only 0.36 % to the total variance, together capturing a cumulative 99.81 % of the variance. PCA revealed a clear separation between strawberry juice (at 0 h) and strawberry juice (at 48 h), indicating that significant changes occurred in the volatile compound composition during this fermentation.

These results indicated that the dominant metabolic alterations during fermentation were primarily captured by PC1, suggesting that specific volatile compounds undergo substantial concentration changes over time during this fermentation. In the initial samples, the tight clustering along PC1 reflected a relatively uniform volatile profile, which indicated that fresh strawberry juice exhibited a stable and homogeneous composition of aroma-related metabolites. Fresh strawberry juice (*at 0 h*) clustered separately from the fermented samples, suggesting that it contained distinct VOC profiles with lower overall levels of volatile compounds. But the sample distribution along PC1 expanded significantly, whereas the dispersion along PC2 increased. This shift suggested that microbial metabolism actively reshaped the volatile profile, leading to increased metabolic heterogeneity. *The strawberry* samples (at 48 h) were grouped closely, indicating that fermentation led to a more uniform VOC composition. The significant shift in the VOCs aligned with the biochemical transformations driven by microbial metabolism, which may include the conversion of aldehydes and sulfur compounds into alcohols, ketones, and other fermentation-related metabolites.

Fermentation led to a marked increase in certain classes of volatile compounds, particularly alcohols and esters, which are known as the key contributors to fruity and floral aromas. The elevated levels of ethanol, isoamyl alcohol, and phenylethyl alcohol suggested an active microbial metabolism of sugars and amino acids through pathways such as Ehrlich's degradation and increased alcohol biosynthesis. Moreover, ester formation was significantly enhanced, with ethyl acetate, isoamyl acetate, and ethyl butanoate showing relatively high concentrations post-fermentation. These esters are known for their sweet, fruity characteristics and are commonly associated with improved sensory attributes in fermented fruit-based products.

Moreover, the contents of aldehydes, such as hexanal and nonanal, decreased after fermentation. This reduction was likely due to microbial enzymatic activity, particularly that of aldehyde dehydrogenases, which convert aldehydes to their corresponding alcohols or acids. Given that aldehydes often impart green, grassy, or fatty aromas, their reduction indicates a more refined and balanced flavor profile in the final product.

Differential changes were noted in terpenes, which are naturally present in strawberries and contribute to floral, citrus, and woody notes. The levels of monoterpenes such as linalool and α-terpineol moderately increased after fermentation, suggesting the potential biotransformation of glycosidically bound precursors into free volatile forms. This metabolic conversion may increase the aromatic complexity of the final product.

The observed increases in alcohols and esters suggested an increase in the desirable fruity and floral notes, which are highly valued in fermented fruit beverages. The reduction in the aldehydes combined with increased terpene bioconversion likely contributes to a smoother, less grassy, and more aromatic sensory profile. These compositional shifts not only improve the perceived quality of fermented strawberry juice but also provide insights for the use of microbial fermentation as a tool for enhancing the functional and sensory properties of fruit-based beverages.

The above findings collectively underscore the significant impact of fermentation on the volatile composition of strawberry juice, revealing how microbial activity selectively modulates the key aroma compounds in fruit juice. The metabolite shifts observed in this study provide valuable insights into the underlying biochemical pathways and for the potential applications for optimizing fermentation conditions to increase product quality in commercial functional beverages.

### 4.6 Flavor volatile compounds

The contents of volatile compounds in fruit juices are important determinants in shaping consumer preferences and sensory perception (Cui et al., 2019; Ratchadaporn Kaprasob, Kerdchoechuen, Laohakunjit, Sarkar, & Shetty, 2017). Strawberries possess a highly complex aroma profile composed of a wide variety of volatile compounds, with about 1000 compounds identified globally (Di Cagno, Filannino, & Gobbetti, 2017). Despite this diversity in aroma compositions, the characteristic strawberry aroma is predominantly a combination of esters, furans, and terpene alcohols, with minute amounts of lactones, aldehydes, alcohols, and sulfur-containing compounds.

**Fig. 7:**
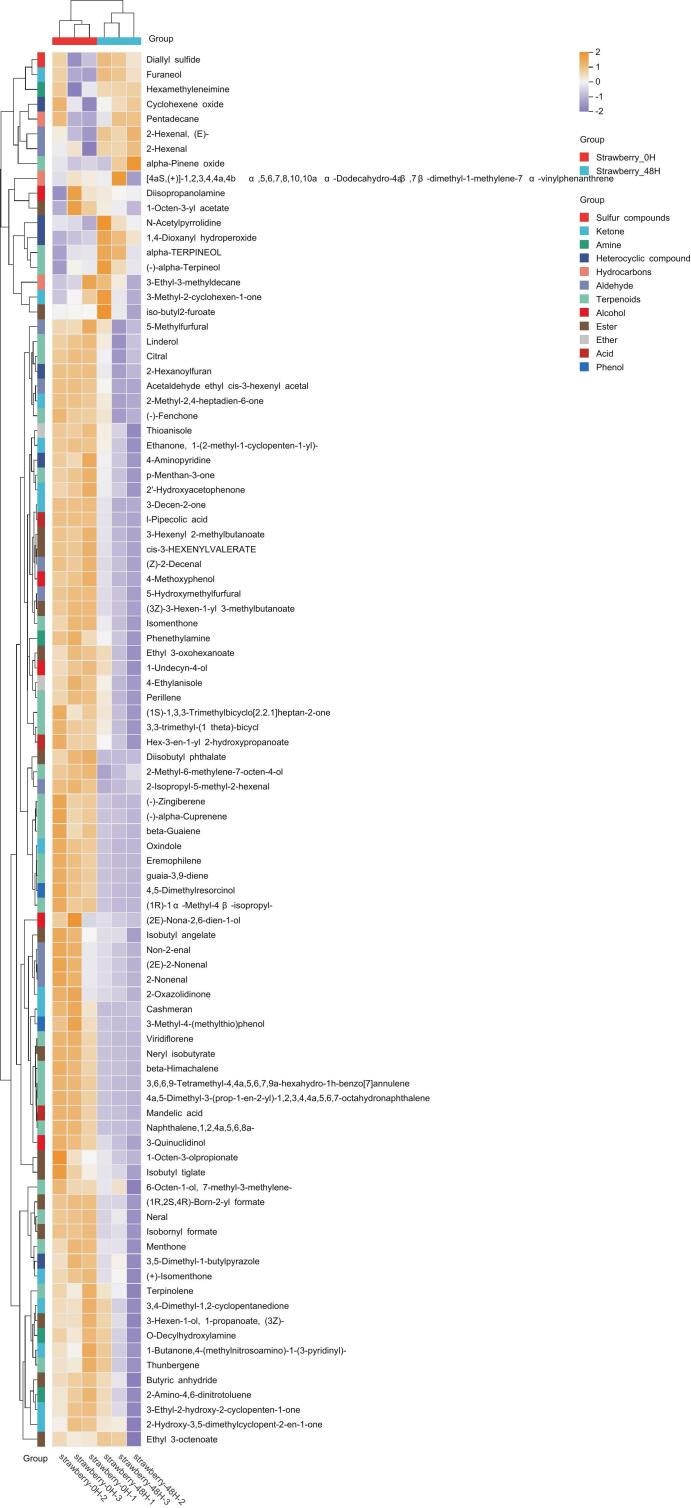
Heat map of flavor compounds identified in strawberry juice with different fermentation time.

This study aimed to comprehensively characterize the volatile aroma profile of strawberries before and after fermentation using HS-SPME-GC–MS. The odor activity value (OAV) for the samples was calculated to assess the contribution of individual volatile compounds in the sample to its overall aroma. To further elucidate the compositional differences, hierarchical cluster analysis and heatmap visualization were employed (Fig. 7). The clustering results revealed distinct grouping patterns, with unfermented samples (at 0 h) forming a single branch and fermented samples (at 48 h) forming a separate branch. This clear segregation indicated substantial compositional changes induced by fermentation.

The heatmap analysis categorized the volatile compounds into different functional classes of alcohols, esters, aldehydes, and terpenoids, highlighting significant metabolic shifts. Notably, the concentrations of alcohols and esters increased substantially post-fermentation, suggesting that these compounds were likely formed through microbial sugar metabolism and lipid degradation pathways. In contrast, the contents of aldehydes and ketones markedly decreased, potentially due to their utilization as intermediate substrates in the microbial metabolic processes. Additionally, the increase in terpenoids was attributed to the activation of microbial secondary metabolism, leading to increased biosynthesis of aromatic compounds.

Odor-active compounds with high levels of OAVs play a dominant role in shaping the sensory attributes of strawberries. While each of these compounds individually contributes to a specific aroma note, their interactions –synergistic or antagonistic – can influence the overall perception of the product. The threshold concentration of each volatile compound is a critical determinant of its sensory impact, with lower thresholds corresponding to stronger olfactory effects. Table 2 presents the key OAVs of the major volatile compounds, highlighting their relative contributions to the aroma profile of strawberries.

**Table 2:** The key OAVs of aroma-active compounds in fermented strawberry juice.

NO.	Compound	Odor Threshold	Aroma Description	0H	48H
1	Mandelic acid	15,200	–	0.33	0.28
2	1-Undecyn-4-ol	0.1	–	0.85	0.79
3	3-Quinuclidinol	380	–	0.28	0.25
4	Diisopropanolamine	0.0085	–	0.19	0.18
5	2-Hexenal	0.017	sweet, almond, fruity, green, leafy, apple, plum, vegetable	128.44	140.68
6	2-Hexenal, (*E*)-	0.0031	green, grassy	677.71	766.54
7	2-Nonenal	0.0001	fatty, green, waxy, cucumber, melon	2718.19	1897.72
8	5-Hydroxymethylfurfural	1000	fatty, buttery, musty, waxy, caramel	0.21	0.19
9	Isobornyl formate	0.15	camphor, musty, medicinal, woody	1.05	0.98
10	2-Hexanoylfuran	1	sweet, fruity, ketonic, green, apricot, peach	1.39	1.26
11	3,5-Dimethyl-1-butylpyrazole	0.017	–	1.62	1.51
12	4-Aminopyridine		–	0.22	0.21
13	Pentadecane	13,000	waxy	0.22	0.26
14	2-Hydroxy-3,5-dimethylcyclopent-2-en-1-one	0.017	strong, caramel	0.28	0.27
15	3-Decen-2-one	0.2	fatty, green, fruity, apple, earthy, jasmin	0.52	0.47
16	Ethanone, 1-(2-methyl-1-cyclopenten-1-yl)-	0.052	–	0.22	0.19
17	Furaneol	0.001	sweet, cotton, candy, caramel, strawberry, sugar	512.71.	618.14
18	Diallyl sulfide	0.1	sulfury, onion, garlic, horseradish, metallic	3.88	4.33
19	Citral	0.1	sharp, lemon, sweet	28.76	25.81
20	6-Octen-1-ol, 7-methyl-3-methylene-	0.15	–	0.51	0.49
21	Neral	1	Sweet, citral, lemon, peel	2.87	2.67

Among the volatiles identified in this study, (E)–2-hexenal presented two distinct odor characteristics: one that was described as “sweet, almond, fruit, green leaves, apple, and plum” with a threshold of 0.017 and the other that was described as “green leaves and grass” with a much lower threshold of 0.0031. During fermentation, the OAV of (E)–2-hexenal increased from 128.44 to 140.68, whereas the OAV for the green, grassy descriptor increased from 677.71 to 766.54. These findings suggested that fermentation intensified the green and grassy attributes of the strawberry aroma. The increase in aldehydes such as (E)-2-hexenal may result from enzymatic oxidation of unsaturated fatty acids. Meanwhile, the overall increase in alcohols could be partially attributed to the reduction of aldehydes via alcohol dehydrogenases, but also likely involves other metabolic pathways such as amino acid degradation and sugar metabolism during fermentation.

The compound 2-nonenal, which imparts a “fat, green, wax, cucumber, melon” aroma and has an extremely low threshold of 0.0001, exhibited a decrease in OAV from 2718.19 to 1897.72 post-fermentation. This reduction indicated a weakening of the waxy, green, and cucumber-like sensory attributes, possibly due to microbial reduction reactions or oxidative degradation. Sweetness is a key determinant of fruit product acceptability. An increase in certain volatile compounds associated with sweet and fruity aromas can, therefore, increase consumer appeal, whereas the presence of acidic or sulfurous notes may negatively impact perception. Citral, a key terpene with a threshold of 0.1, is responsible for sharp, lemonistic, and mildly sweet notes. The OAV of citral in this study decreased from 28.76 to 25.81, indicating a slight reduction in the citrus aroma. However, owing to its initially high intensity, the overall sensory impact remained significant. The decrease in citral may be attributed to microbial reduction or chemical degradation into non-volatile derivatives. Diallyl sulfide, a sulfur-containing compound that imparts an onion-like and garlic-like aroma, exhibited OAV increasing from 3.88 to 4.33. The increase in the OAVs of sulfur compounds suggested microbial metabolism of sulfur-containing amino acids, such as methionine and cysteine, leading to the formation of volatile sulfur compounds. Although present at relatively low concentrations, these compounds can contribute to the off-flavors and potentially affect consumer acceptance. Furaneol is a key contributor to the characteristic caramel-like and sweet strawberry aroma, and its OAV substantially increased from 512.71 to 618.14 in this study. This enhancement strengthened the sweet and fruity attributes of the fermented product, making it more aromatic and appealing. The increase in furaneol may be linked to the enzymatic hydrolysis of glycosidic precursors during fermentation. Neral, another terpene with lemon-like characteristics, exhibited a minor decrease in OAV from 2.87 to 2.67. This slight reduction, however, did not significantly impact the overall aroma profile of the fermented product.

The fermentation process significantly modified the volatile compound profile of strawberry juice. The increased concentrations of key odorants, such as (E)–2-hexenal and furaneol, intensified the green, grassy, sweet, and fruity sensory attributes of the juice. Conversely, the reduction in Citral and Neral contributed to a decrease in the acidic, lemon-like notes. Additionally, the increase in Diallyl sulfide levels introduced subtle sulfurous nuances, which could affect the overall aroma perception.

Interestingly, **diallyl sulfide**, a volatile more commonly associated with garlic and onion, was also detected in our fermented strawberry juice. While its presence in fruit fermentations is rare, previous studies have identified diallyl sulfide in lactic acid-fermented vegetable matrices, such as model kimchi(Hong, Lee, Kim, & Ahn, 2016). Additionally, sulfur-containing volatiles have been occasionally reported in fermented vegetables We propose that L. *fermentum* FUA033 may metabolize sulfur-containing amino acids to yield diallyl sulfide under specific fermentation conditions. Were also detected in our study, contributing to the complex aroma profile.

These metabolic transformations were driven primarily by microbial enzymatic activities, including aldehyde reduction, esterification, and sulfur metabolism. The formation of these new volatile compounds and the conversion of precursor molecules contributed to a more complex and mature aroma profile. The overall effect of fermentation was the enhancement of fruity and sweet characteristics while modulating the undesirable attributes, ultimately leading to a more balanced and appealing strawberry aroma.

### 4.7 Analysis on the sensory properties of the fermented strawberry juice

**Fig. 8:**
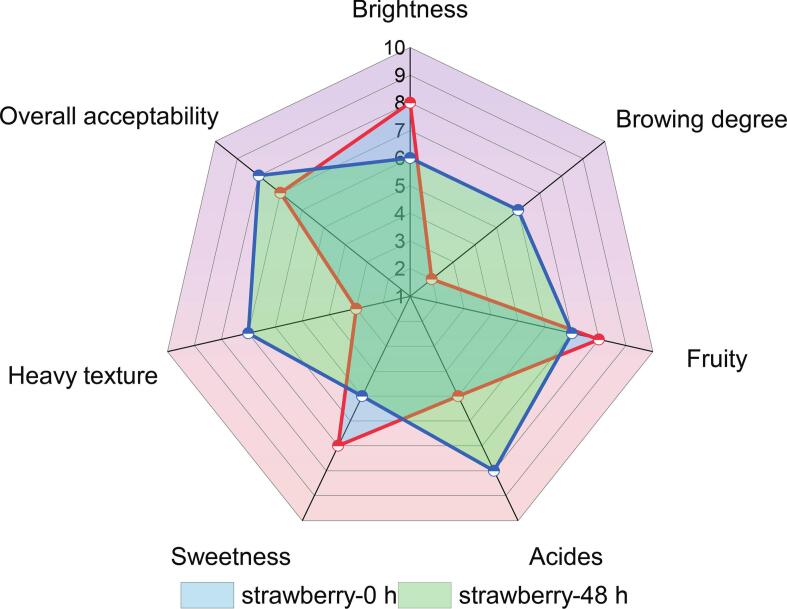
Sensory radar chart for the mean values of the sensory attributes of the strawberry juice with different fermentation time.

In this study, the sensory characteristics of strawberry juice before and after 48 h of fermentation were systematically evaluated through descriptive analysis (DA), focusing on sweetness, acidity, fruity aroma, browning degree, brightness, heavy texture, and overall acceptability (Fig. 8). Fermentation induced significant alterations in several organoleptic attributes. Sweetness was markedly reduced in the 48-h fermented sample compared to the fresh juice (0H), consistent with microbial consumption of sugars during lactic acid fermentation. In contrast, acidity scores increased significantly post-fermentation, reflecting the accumulation of organic acids such as lactic and acetic acids. Fruity aroma exhibited a moderate decrease, possibly due to the degradation or transformation of volatile esters during the fermentation process. Visually, the fermented sample showed increased browning degree and slightly reduced brightness, suggesting pigment degradation and Maillard-type reactions associated with fermentation. Notably, the “heavy texture” attribute was enhanced in the 48H sample, indicating a richer and more viscous mouthfeel, potentially due to microbial exopolysaccharide production or compositional changes in the juice matrix. Interestingly, despite the loss of sweetness and fresh fruity notes, the overall acceptability of the fermented sample was higher than that of the unfermented juice. This suggests that the complex flavor profile and improved texture resulting from fermentation were positively perceived by the panelists. These results confirm that lactic acid fermentation significantly modulates the sensory profile of strawberry juice, reducing sweetness and brightness while enhancing acidity, texture, and overall palatability.

## 5 Conclusion

Fermentation with *L. fermentum* FUA033 supported vigorous bacterial growth in strawberry juice and markedly reshaped its chemical composition and sensory traits. These results confirmed that L. *fermentum* FUA033 can effectively grow in strawberry juice and significantly enhance its biochemical composition and sensory properties. Compared to that of unfermented juice, the fermentation process led to a notable increase in the TPC, TFC, and SSC while also enriching the volatile compound profile. These changes contributed to a more complex and desirable aroma. Additionally, compared to the unfermented control, the fermented fermentation process significantly increased the total phenolic, flavonoid, and soluble- solid contents while enriching the spectrum of volatile compounds, thereby generating a more complex and appealing aroma. The antioxidant capacity of the fermented beverage surpassed that of the raw juice, underscoring its enhanced functional potential. Crucially, EA underwent efficient microbial conversion to Uro-A, highlighting fermentation as a viable, solvent-free approach for the industrial production of this bioactive metabolite. These findings underscore the potential industrial application potential of microbial fermentation in Uro-A production. In summary, fermentation with L. *fermentum* FUA033 enhances the bioactivity and aromatic complexity of strawberry juice by modifying its flavonoid, polyphenol, and volatile compound contents. Moreover, descriptive sensory analysis revealed that the fermented sample exhibited significantly higher scores in acidity, mouthfeel richness, and overall acceptability compared to the unfermented control. These improvements in organoleptic quality further support the potential consumer appeal of fermented strawberry juice.

Overall, *L. fermentum* FUA033 fermentation improves the bioactivity and flavor complexity of strawberry juice. These findings provide a reference framework for optimizing similar fruit-based functional beverages.

## CRediT authorship contribution statement

**Qing Wu:** Writing – review & editing, Writing – original draft, Methodology, Investigation, Data curation, Conceptualization. **Wenyue Ma:** Investigation, Data curation. **Shu Liu:** Supervision. **Fandi Zhou:** Investigation. **Hao Wu:** Formal analysis. **Xiangyuan Wang:** Supervision. **Guang Yang:** Writing – review & editing, Supervision. **Yaowei Fang:** Writing – review & editing, Supervision, Project administration, Funding acquisition.

## Funding

This work was supported by the 10.13039/501100012246Priority Academic Program Development of Jiangsu Higher Education Institutions, the Key Natural Science Foundation of the Jiangsu Higher Education Institutions of China (20KJA550001), Project “333” of 10.13039/501100002949Jiangsu Province, and the College Students' Innovation and Entrepreneurship Training Program of 10.13039/501100002949Jiangsu Province (SY202311641638001).

## Declaration of competing interest

The authors declare that they have no known competing financial interests or personal relationships that could have appeared to influence the work reported in this paper.

## Data Availability

The data that has been used is confidential.
